# CORRELATION OF THE SAGITTAL BALANCE WITH POSTURAL ANALYSIS OF THE PELVIS AND LUMBAR SPINE

**DOI:** 10.1590/1413-785220243201e274089

**Published:** 2024-03-22

**Authors:** Marília Simões Lopes Quintana, Angelica Castilho Alonso, Natália Mariana Silva Luna, Jessica Paulino da Silva, Matheus Henrique dos Santos Lino, Guilherme Carlos Brech, Júlia Maria D’Andrea Greve

**Affiliations:** 1 Universidade de São Paulo (FMUSP), School of Medicine, Orthopedics and Traumatology Institute of the Hospital das Clínicas, Laboratory Study of Movement, São Paulo, SP, Brazil.; 2 Universidade São Judas Tadeu (USJT), Graduate Program in Aging Sciences, São Paulo, SP, Brazil.

**Keywords:** Spine, Software, Rehabilitation, Posture, Coluna Vertebral, Software, Reabilitação, Postura

## Abstract

**Objective:**

Evaluate and correlate the sagittal balance parameters with the postural of the pelvis and lumbar spine.

**Methods:**

80 individuals of both sexes, aged between 20 and 35 years, were evaluated. Biophotogrammetry was done with the SAPO software program. Measurements of the sagittal balance parameters were obtained by analyzing a lateral view panoramic radiography of the vertebral column, in which the anatomical points of reference were digitally marked. The calculation of the angles was done automatically by the Keops program.

**Results:**

In Keops assessment, 17.5% of the sample had high pelvic incidence angles (> 60°), 31.5% had low pelvic incidence angles (< 45°), and 51.2% had medium pelvic incidence angles (between 46° and 59°). SAPO showed 12,5% lordosis, 40% retroversion, and 47,5% normal curvature. In the right lateral view, pelvic incidence angle had a moderate and positive correlation with vertical alignment of the trunk and with vertical alignment of the body, and a negative and moderate correlation with horizontal alignment of the pelvis.

**Conclusion:**

Differences were found between vertical alignment measurements from the postural evaluation system (SAPO). A positive correlation was found between PI from Keops and pelvic anteversion from SAPO. ***Level of Evidence II; Prospective Study***.

## RESUMO

**Objetivo:**

Avaliar e correlacionar o equilíbrio sagital com parâmetros posturais da pelve e coluna lombar

**Métodos:**

Foram avaliados 80 indivíduos de ambos os sexos, com idade entre 20 e 35 anos. A biofotogrametria foi realizada com o software SAPO. As medidas dos parâmetros do equilíbrio sagital foram obtidas pela análise de uma radiografia panorâmica em perfil da coluna vertebral, na qual os pontos anatômicos de referência foram marcados digitalmente. O cálculo dos ângulos foi feito automaticamente pelo programa Keops.

**Resultados:**

Na avaliação Keops, 17,5% da amostra apresentavam ângulos de incidência pélvicos altos (> 60°), 31,5% tinham ângulos de incidência pélvicos baixos (< 45°) e 51,2% apresentavam ângulos de incidência pélvicos médios (entre 46° e 59°). O SAPO apresentou 12,5% de lordose, 40% de retroversão e 47,5% de curvatura normal. Na vista lateral direita, o ângulo de incidência da pelve apresentou correlação moderada e positiva com o alinhamento vertical do tronco e com o alinhamento vertical do corpo e negativa e moderada com o alinhamento horizontal da pelve.

**Conclusão:**

Foram encontradas diferenças entre as medidas de alinhamento vertical do sistema de avaliação postural (SAPO). Uma correlação positiva foi encontrada entre IP de Keops e anteversão pélvica de SAPO. ***Nível de Evidência II; Estudo Prospectivo***.

## INTRODUCTION

Posture acquisition occurs throughout life and is affected by constitutional, environmental, and lifestyle factors. Postural deviations can affect the musculoskeletal structures and cause an abnormal displacement of the center of mass over the support base, generating pain, joint instability, muscular weakness and functional deficiencies,^1^ in addition to altering the load and pressure distribution on the joint surfaces, leading to joint degeneration and inadequate muscular tensions.^2^ Making a good assessment is the first step in treating alterations of body alignment and posture. One of the most used forms of assessment is biophotogrammetry, which can be done with the Postural Evaluation System (SAPO), a software program that measures horizontal and vertical angles and distances between body segments using photographs with luminescent markers placed in predetermined regions of the body.^3^ Assessment of the sagittal plane of balance with a three-dimensional view is a recent development that has been used to evaluate and plan surgical procedures for stabilization of the spine. Sagittal balance depends on the morphological and spatial parameters of the pelvis, hip and spine, measured with a panoramic radiography of the spine. The main parameters used are the pelvic incidence angle (PI),^4^ defined as the angle between a line perpendicular to the midpoint of the sacral plateau and another line connecting this point to the central axis of the femoral head. It is an anatomical parameter, constant, immutable and exclusive of each individual, which does not depend on age and orientation of the pelvis and which is consolidated after bone growth ends.^5,6^ In addition to PI, the sacral slope angle (SS) and the pelvic version angle or pelvic balance angle (PV) are also used in the diagnosis, evolutionary prognosis and treatment of spinal disorders. Sagittal balance parameters are objective measures that open a new perspective in the evaluation and treatment of postural alterations, improving rehabilitation and training programs by making them more specific and individualized.

The goal of this study was to evaluate the sagittal balance parameters measured by the software program *Keops* and the postural parameters in the right and left side views measured by the SAPO program, and to look for correlations among the parameters measured by *Keops* and between those and results from biophotogrammetry of the pelvis and lumbar spine.

## METHODS

### Design, local and ethics

This is a cross-sectional study performed at the Laboratory for the Study of Movement (LEM) of the Institute of Orthopedics and Traumatology of Hospital das Clínicas from the Medical School of the University of São Paulo (HC FMUSP) approved by the Ethics Research Committee (protocol number: 0466/12).

### Participants

A total of 80 individuals were evaluated, being 46 females and 34 males, with a mean age of 25 ± 4.1 years. The inclusion criteria were: Having a level of cognition sufficient to understand the procedures and follow given instructions, sedentary individuals, according to the International Physical Activity Questionnaire (IPAC), individuals with BMI < 30, absence of disease/traumas or *sequelae* that compromise the locomotor system (musculoskeletal and nervous system) and absence of diseases of the vestibular system or compromised balance. The exclusion criteria were not being able to perform the tests.

### Procedures

All individuals agreed to participate in the study by reading and signing the informed consent form. After that they answered a questionnaire with personal information, socio-demographic.

### Assessments

**Assessment 1:** Panoramic radiography of the vertebral column in orthostatic position with the arms flexing in front of the body. The panoramic images were submitted to the *Keops* program after identifying the anatomical points used to measure the sagittal balance parameters: PI, PV, and SS.

**Assessment 2:** Postural evaluation with the Postural Evaluation System (SAPO). Styrofoam markers were placed in the anatomical points of the trunk and limbs: acromion, anterior superior iliac spine, posterior superior iliac spine, greater trochanter, and lateral malleolus. Images from the right and left lateral views were used: vertical alignment of the trunk, vertical alignment of the body, and horizontal alignment of the pelvis.

### Statistical Analysis

The normality and homogeneity of variance were confirmed by the Komorov-Smirnov test and levene test, respectively. The data were presented by means of means, standard deviation (SD). Pearson’s correlation was used to relate the dependent variables: lumbar lordosis angles, thoracic cifosis, pelvic angle of incidence, pelvic inclination and sacral inclination with independent variables (SAPO angles): vertical alignment of the trunk, vertical alignment of the body and horizontal alignment of the pelvis. For the entire analysis, the statistical software SPSS (Statistical Package for Social Science) version 22.0 for Windows was used and a significance index of p ≤ 0.05 was adopted.

## RESULTS

Table 1 shows the angles measured by the Keops and SAPO software programs. In the SAPO program, the right and left lateral views were analyzed and in the Keops program, the pelvic incidence angle was measured in 80 individuals of both sexes.

**Table 1:** Angles (degrees) measured by the Keops and SAPO programs for postural evaluation (n = 80).

Angle	Average (SE)
KEOPS	
Pelvic Incidence (°)	51.2 (8.1)
Sacral Slope (°)	41.1 (6.2)
Pelvic Tilt (°)	10.3 (6.0)
Lumbar lordosis angle (°)	60.3 (11.3)
Thoracic kyphosis angle (°)	37.2 (12.3)
**SAPO: right side view**	
Vertical alignment of the trunk (°)	-2.5 (3.2)
Hip-trunk and thigh angle (°)	-5.3 (6.9)
Vertical alignment of the body (°)	1.4 (2.0)
Horizontal alignment of the pelvis (°)	-7.1 (12.4)
**SAPO: left side view**	
Vertical alignment of the trunk (°)	-1.5 (2.8)
Hip-trunk and thigh angle (°)	-4.5 (6.4)
Vertical alignment of the body (°)	2.7 (1.4)
Horizontal alignment of the pelvis (°)	-6.3 (1.9)

Fourteen (17.5%) had a high pelvic incidence angle, above 60° (63.8 ± 4.0°); 41 (51.2%) had a moderate pelvic incidence angle between 45-59° (52.0 ± 3.4°) and 25 (31.2%) had a low pelvic incidence angle, under 45° (42.8 ± 1.9°). The SAPO program measurements resulted in 10 (12.5%) individuals with pelvic anteversion, 32 (40%) with pelvic retroversion, and 38 (47.5%) with neutral pelvis, assessed by the horizontal alignment of the pelvis in the right and left lateral views. (Table 2)

**Table 2:** Distribution of the subjects according to the pelvic incidence angles (PI) measured by the Keops program and pelvis position assessed by SAPO (n = 80).

PI	High (> 60°)	Medium (45-59°)	Low (< 45°)
	14	41	25
**Pelvis**	**Anteversion**	**Neutral**	**Retroversion**
	10	38	32

PI: Pelvic incidence angle.

We looked for correlations among the angles measured by the *Keops* software program (Table 3). The sacral inclination and lumbar lordosis angles showed a positive and moderate correlation; the pelvic incidence angle showed a positive and weak correlation with the lumbar lordosis angle, and the thoracic kyphosis angle showed a positive and weak correlation with the lumbar lordosis angle. Tables 4 and 5 show the correlations between measurements of the Keops and SAPO software programs: Pelvic incidence angle showed a moderate and positive correlation with vertical alignment of the trunk, and a negative and moderate correlation with horizontal alignment of the pelvis. Horizontal alignment of the pelvis showed a moderate and negative correlation with lumbar lordosis angle in the right lateral view. In the left lateral view, horizontal alignment of the pelvis showed a weak and negative correlation with pelvic incidence angle, and a moderate and negative correlation with lumbar lordosis angle.

**Table 3:** Correlation values (r) between the pelvic incidence angle, pelvic tilt angle, and sacral slope angle with the thoracic kyphosis angle, and lumbar lordosis angle measured by the Keops program (n = 80).

Angles	Lumbar lordosis angle	Thoracic kyphosis angle
	r(p)	r(p)
Pelvic incidence	0.546 (p ≤ 0.001)*	0.057 (0.615)
Sacral slope	0.541 (p ≤ 0.001)*	0.010 (0.926)
Pelvic tilt	0.122 (0.283)	0.028 (0.804)
Lumbar lordosis angle	–	0.334 (0.002)*
Thoracic kyphosis angle	0.334 (0.002)*	-

Pearson’s coefficient

* p ≤ 0.05.

**Table 4:** Correlation values (r) between the Keops sagittal balance variables and the SAPO postural variables in the right lateral view for the group of subjects (N = 80).

Variables	VAT	HTTA	VAB	HAP
	r(p)	r(p)	r(p)	r(p)
Pelvic incidence	0.638 (0.053)*	0.166 (0.141)	0.078 (0.491)	-0.496 (p ≤ 0.001)*
Sacral slope	-0.031 (0.786)	- 0.042 (0.714)	0.049 (0.668)	-0.288 (0.010)
Pelvic tilt	0.000 (0.999)	-0.181 (0.108)	0.044 (0.696)	-0.250 (0.025)
Lumbar lordosis angle	-0.067 (0.557)	-0.136 (0.228)	-0.043 (0.707)	-0.376 (p ≤ 0.001)*
Thoracic kyphosis angle	0.064 (0.574)	-0.047 (0.678)	0.032 (0.267)	0.031 (0.787)

Pearson’s coefficient (R)

* p ≤ 0.05. Legend: VAT - vertical alignment of the trunk; HTTA: hip-trunk and thigh angle; VAB: vertical alignment of the body; HAP: horizontal alignment of the pelvis.

**Table 5:** Correlation values (r) between the Keops sagittal balance variables and the SAPO postural variables in the right lateral view for the group of subjects (N = 80).

Variables	VAT	HTTA	VAB	HAP
	r(p)	r(p)	r(p)	r(p)
Pelvic incidence	0.638 (0.053)*	0.166 (0.141)	0.078 (0.491)	-0.496 (p ≤ 0.001)*
Sacral slope	- 0.031 (0.786)	- 0.042 (0.714)	0.049 (0.668)	-0.288 (0.010)
Pelvic tilt	0.000 (0.999)	-0.181 (0.108)	0.044 (0.696)	-0.250 (0.025)
Lumbar lordosis angle	-0.067 (0.557)	-0.136 (0.228)	-0.043 (0.707)	-0.376 (p ≤ 0.001)*
Thoracic kyphosis angle	0.064 (0.574)	-0.047 (0.678)	0.032 (0.267)	0.031 (0.787)

Pearson’s coefficient (R)

* p ≤ 0.05. Legend: VAT - vertical alignment of the trunk; HTTA: hip-trunk and thigh angle; VAB: vertical alignment of the body; HAP: horizontal alignment of the pelvis.

## DISCUSSION

High pelvic incidence angles are correlated with increased pelvic anteversion or increased lordosis. The sagittal balance measurements allow us to quantify the degree of lumbar lordosis and pelvic version, thus providing objective measurements which can be used to evaluate the results of treatment programs for disorders of the spine. Postural assessment can be done by physical examination and by specific quantitative tests, both of which are subjective and poorly reproducible. A panoramic radiography of the spine, aided by anatomical reference points, improves the assessment of normal and pathological postures.

Sagittal balance variables, measured by digital evaluation of the panoramic radiography of the spine, are not often used in physiotherapy, and we could not find in the literature studies that used or compared this method of evaluation with biophotogrammetry and/or other commonly used evaluation methods. Sagittal balance variables have been valued^5–9^ in the evaluation and treatment of disorders of the spine, especially in surgical treatments.

Weinberg et al.^8^ found a higher PI among black people. In the present study, although the influence of race was not evaluated, there was a predominance of low pelvic incidence angle, a result that would not be expected for the Brazilian population, which has a high degree of racial miscegenation between whites and blacks. This is something to be elucidated in future studies with larger samples. Some authors report no significant differences between men and women regarding the pelvic incidence, sacral slope and pelvic tilt angles,^9–12^ but according to Sudhir et al.^11^ the pelvic incidence angle is larger in women. In this study, no difference was found between the sexes: 46 women had a PI of 50.1 ± 7.8°, and 34 men had a PI of 52.7 ± 8.4°, in agreement with some authors.^9–12^

The pelvic tilt and sacral slope angles were 10.3 ± 6.0° and 41.1 ± 6.2°, respectively, within the normal range (10°-25° for pelvic tilt, and 30°-50° for sacral slope)^13,14^ as expected for the study population. Rogala et al.^15^ reported that high pelvic tilt angles are associated with pelvic retroversion, constituting a compensatory measure for maintaining sagittal balance. The lumbar lordosis and thoracic kyphosis angles were 60.3 ± 11.3° and 37.2 ± 12.3°, respectively, In the right and left lateral view assessment done by SAPO, 47.5% of the sample had pelvic anteversion (-12.7 ± 3.7° in the right side view, and -10.9 ± 7.4, in the left side view) within the physiological values of a pelvis in neutral position. Lumbar lordosis with anteversion (-15.7 ± 1.3° in the right side view, and -15.2 ± 1.6°, in the left side view) was found in 12.5% of the sample. Retroversion (7.2 ± 1.6° in the right side view, and 7.3 ± 1.6° in the left side view) was found in 40% of the sample.

Souza et al.^16^ assessed the reliability of the horizontal alignment of the pelvis and found values that are in agreement with the present study, with a large variation but good reliability, however, he also acknowledged the difficulties involved in adequately collecting the SAPO parameters.

Results from the *Keops* software program are in agreement with those from SAPO for the current sample. There is a predominance of moderate pelvic incidence angles (*Keops*), and physiological anteversion (SAPO), followed by low incidence angles (*Keops*) and retroversion (SAPO), and finally by individuals with high incidence angles (*Keops*) and anteversion of the pelvis (SAPO). The two methods converged on the results, a fact that corroborates the reliability of both.

Nery^17^ reported that the SAPO program is a good tool for postural evaluation, but had reservations regarding the lateral views. Souza et al.^16^ reported that horizontal alignment of the pelvis and vertical alignment of the body, as measured by SAPO, are highly reliable, but emphasized that vertical alignment of the trunk and hip angle are not reliable, mainly due to the difficulties involved in properly positioning the markers.

Ferreira et al,^7^ in a study aimed at validating the SAPO software program, reported that horizontal alignment of the pelvis, vertical trunk and body alignment, and hip angle are reliable measurements. The four measurements are within the normal range reported in the literature, according to which, pelvic anteversion can range from -10 to -15°, however, the large variation in horizontal pelvic alignment makes it difficult to establish physiological parameters. The software program *Keops*, is based on an objective measurement, i.e., a panoramic radiography of the vertebral column, and it also associates large negative anteversion angles with high pelvic incidence angles and lumbar lordosis. Pelvic incidence angle has a moderate and positive correlation with vertical alignment of the trunk and the body, showing that the larger the anterior tilt of the body, the larger the angle of pelvic incidence, due to the need of maintaining postural balance in orthostatism. In the right lateral view, a moderate and positive correlation between PI and horizontal alignment of the pelvis was found, showing that the higher the PI and lumbar lordosis, the greater the anteversion of the pelvis, with the angles of horizontal alignment of the pelvis becoming more negative. These data were not completely confirmed, since in the left lateral view the correlation was positive but weak. The imprecision of the lateral measurements in SAPO may justify the difference between the right and left lateral views. The best correlation found was between PI and horizontal alignment of the pelvis, a fact which should be expected according to the literature.^5,6,10,7,12^

There is a tendency of associating PI with the position of the pelvis and curves of the lumbar and thoracic spine, however, there is a range of small variations in the posture acquisition process which may justify the moderate and weak correlation found between the variables from Keops and SAPO.

PI is the determining parameter of sagittal balance, because it is individual, constant, and remains with the individual during their lifetime,^9,10,14^ thus directly interfering in the spinopelvic relation. However, acquisition of the postural pattern, which takes place throughout the life of an individual, can be influenced by other factors besides the PI angle, which leads to variations in pelvis positioning and spinal curvature.

PI alone cannot be used as an indicator of pelvis positioning and spinal curvature, since an individual may create different strategies to maintain sagittal balance. PI is a good guiding parameter, but other variables need to be considered to better understand an individual’s posture.

Sagittal balance variables are useful for surgeons and rehabilitation teams to better evaluate and treat spinal disorders, mainly due to its three-dimensional character.

The SAPO program variables used in this study were those that measure the same regions composed by *Keops*: position of the pelvis and curvatures of the spine. SAPO is a two-dimensional evaluation and therefore may be insufficient to evaluate the spinopelvic relation.^7^ SAPO is a useful tool which complements clinical evaluation, due to standardization of the measurements,^17^ however, an objective tool like the software program *Keops*, which is based on radiography, improves the objective evaluation and has a lower likelihood for errors. The photographic technique, which uses markers placed at specific points on the body, can lead to positioning errors and measurement errors, especially in lateral views.^17^

The association of three-dimensional measurements, such as the evaluation performed by the *Keops* program, can improve postural assessment and physiotherapeutic interventions.

## CONCLUSIONS

The majority of the study population was within the normal range, having a moderate pelvic incidence angle (PI) and neutral pelvic anteversion.

Differences were found between the right and left vertical alignment measurements from the postural evaluation system (SAPO).

No correlations were found among the three-dimensional variables of sagittal balance from the Keops program.

A positive correlation was found between PI from Keops and pelvic anteversion from SAPO.
